# Genomic characterization of mutant laboratory mouse strains by exome sequencing and annotation lift-over

**DOI:** 10.1186/s12864-015-1548-7

**Published:** 2015-05-06

**Authors:** Sophia Derdak, Sibylle Sabrautzki, Martin Hrabě de Angelis, Marta Gut, Ivo G Gut, Sergi Beltran

**Affiliations:** Centro Nacional de Análisis Genómico, Parc Científic de Barcelona – Torre I, Baldiri Reixac, 4, 08028 Barcelona, Spain; Helmholtz Zentrum München, German Research Center for Environmental Health (GmbH), Institute of Experimental Genetics and German Mouse Clinic, Ingolstädter Landstr.1, 85764 Neuherberg, Germany; Member of German Center for Diabetes Research (DZD), Neuherberg, Germany; Technische Universität München, Lehrstuhl für Experimentelle Genetik, 85350 Freising-Weihenstephan, Germany

**Keywords:** Mutagenesis, Mouse strains, Exome sequencing, Sequence homology, Genomic variants, Annotations, Phenotype

## Abstract

**Background:**

Exome sequencing has become a popular method to evaluate undirected mutagenesis experiments in mice. However, the most suitable mouse strain for the biological model may be relatively distant from the standard mouse reference genome. For pinpointing causative variants, a matching reference with gene annotations is essential, but not always readily available.

**Results:**

We present an approach that allows to use murine Ensembl annotations on alternative mouse strain assemblies. We resolved ENU-induced mutation screening for 8 phenotypic mutant lines generated on C3HeB/FeJ background aligning the sequences against the closely related, but not annotated reference of C3H/HeJ. Variants occurring in all strains were filtered out as specific for the C3HeB/FeJ strain but unrelated to mutagenesis. Variants occurring exclusively in all individuals of one mutant line and matching the inheritance model were selected as mutagenesis-related. These variants were annotated with gene and exon names lifted over from the standard murine reference mm9 to C3H/HeJ using megablast. For each mutant line, we could restrict the results to exonic variants in between 1 and 23 genes.

**Conclusions:**

The presented method of exonic annotation lift-over proved to be a valuable tool in the search for mutagenesis-derived coding genomic variants and the assessment of genotype-phenotype relationships.

## Background

ENU mutagenesis is a popular method to introduce single nucleotide mutations in the mouse genome [1]. Owing to availability or experimental preferences, different inbred mouse strains are submitted to this procedure. Before massive sequencing technologies became available, phenotype-related ENU-induced mutations were short-listed in a lengthy procedure of out-crossing and meiotic mapping that identified linkage chromosomes, or finer linkage regions of approximately 20 MB. Since the bulk of phenotype-causing mutations identified to date after ENU mutagenesis are exonic, exome sequencing would be a welcome method to speed up this process, provided that it allows to narrow down sufficiently the list of variants that will be then submitted to experimental validation.

For the large-scale Munich ENU mutagenesis project [2] the C3HeB/FeJ (C3H) strain was chosen due to the observed high mutation loads and fertility rates following ENU treatment in early pilot studies. The strain showed a good tolerance of the mutagen with low mortality and high fertility rates [2-4]. Moreover, once archived by cryo-preservation good results for in vitro fertilization and embryo transfer were observed [5,6].

Whole genome sequencing can be performed without existing reference sequence nor functional annotations, and exome sequencing for almost any mouse strain used in the lab could be performed using standard mouse exome capture kits based on the biological similarity of the captured sequences and a moderate mismatch tolerance of the capture probes [7]. However, high quality alignments and variant calling depend on a quality reference sequence. If the genome of the sample is very different from the reference sequence, not only a much higher number of variants will be detected, but many reads will not be mapped altogether due to a limited number of mismatches allowed by the mapping algorithm. Furthermore, phenotypes are most commonly related to altered or dysfunctional proteins; therefore, functional genome annotations such as gene and exon boundaries are required to classify variants and to select possible causative mutations. The standard mouse reference genome, UCSC mm9 (NCBI 37 [8]), has been thoroughly annotated in this respect, and there is an effort to generate genome assemblies for several alternative mouse strains [9]. However, strain-specific reference assemblies feature different chromosomal coordinates and only a limited extent of annotation. Considering these restrictions and requirements, it is advisable to align the data to the reference closest to the strain used in the experiment, and lift over exon coordinates from the well-annotated mm9 assembly to the alternative reference, thus achieving a strain-specific, roughly annotated genotyping result. We performed this procedure for sequences of 8 ENU-mutated lines of C3HeB/FeJ mice aligned to the alternative reference assembly for C3H/HeJ and annotated by exon lift-over from mm9.

## Results

For a direct comparison, we mapped the raw reads from the exome sequencing experiment to both the mm9 and the C3H/HeJ reference sequences. The mapping statistics showed a slight, but consistent improvement of alignment against the C3H/HeJ reference as compared to the mm9 reference: As an example, for the sequencing units for the 4 samples of line 1, 79.6% of reads were uniquely mapped to mm9; the error rate was 0.63% on average for read 1 and 0.83% on average for read 2.

Meanwhile, 80.16% of the reads were uniquely mapped to C3H/HeJ; the error rate was 0.54% on average for read 1 and 0.77% on average for read 2.

**Figure 1:**
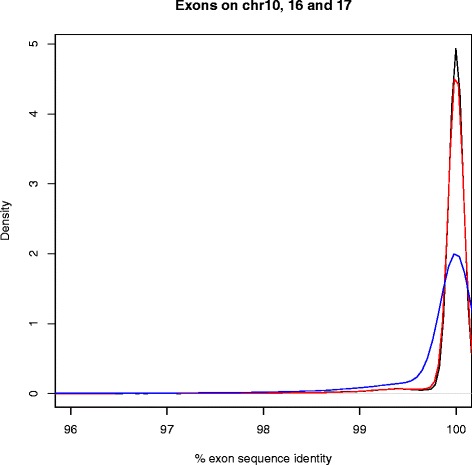
Density plot of % sequence identity of the mm9 exons on chromosomes 10 (black), 16 (red) and 17 (blue) when megablast-ed against the C3H/HeJ reference sequence.

The hits obtained from exon lift-over using megablast showed an average sequence identity of 99.82%, the exons on chr17 showing the lowest identity (min 72.73%, mean 99.69%) and the exons on chr10 and chr16 showing the highest identity (99.92%), as shown in Figure 1.

**Figure 2:**
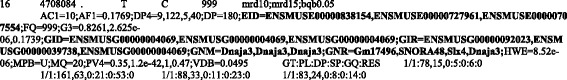
Example variant position as annotated in vcf format: The only private, exonic position in Line 1, where all 4 individuals show an homozygous alternative genotype. Annotations from the exon lift-over appear in the INFO section of the vcf file: EID Ensemble exon id, GID Ensemble gene id for the exons in EID, GIR Ensembl gene id for all genes overlapping the position, GNM associated gene names for the exons in EID, GNR associated gene names for all genes overlapping the position.

Variant call files annotated with the information obtained from exon lift-over were used for selecting candidate variant positions (Figure 2).

Applying the filtering strategy described under Materials and Methods, we were able to isolate between 1 and 23 variant positions per line, including both single nucleotide variants and small insertions/deletions (Table 1). These numbers were reduced to between 0 and 16 positions per line after selecting only positions in exons that did not have any other variant and only positions with ENU-type base changes.

**Table 1:** **Selected variant positions**

**Line code and phenotype class**	**Expected mode of inheritance**	**Selected genotype in vcf file**	**Number of animals sequenced**	**Private variant positions**	**Variant positions in exons**	**Variant positions in exons (one per gene)**	**Variants in linkage region**	**Variant positions with ENU- type base changes**	**Variant positions in concordance with phenotype**
Line 1 body weight	Recessive	1/1	4	3	1	1	No region available	1	No position confirmed
Line 2 body size	Dominant	0/1	4	9	3	3	No region available	2	1 (confirmed by Sanger)
Line 3 hormone level	Dominant	0/1	4	9	3	3	No region available	2	1 position in line for validation
Line 4 enzymatic level	Recessive	1/1	4	1	1	1	1	0	1 position related to the phenotype
Line 5 hormone level	Dominant	0/1	2	136	10	10	No region available	6	4 positions related to the phenotype
Line 6 dental phenotype	Dominant	0/1	2	176	12	10	No region available	4	1 (confirmed by Sanger, manuscript under review)
Line 7 body weight	Dominant	0/1	2	64	14	12	1	5	1 (confirmed by Sanger)
Line 8 body size	Dominant	0/1	2	100	23	23	1	16	No position confirmed

The table summarizes mutant line information, genotypes of interest, numbers of variants at several stages of filtering, numbers of variants with one of the predominant ENU-induced base changes, and numbers of variant positions inside genes relating to the phenotype of each line.

In 3 of the 8 lines, one of these positions could be confirmed by Sanger sequencing in at least ten mutant offspring as the underlying mutation responsible for the phenotype. These mutations were not found in wild-type littermates. In 3 further lines, at least one of these positions may be related to the phenotype of the line via the described gene function, and validation of the genotype-phenotype relationship is ongoing in these lines. For the remaining 2 lines, the detected variants did not affect genes obviously related to the phenotype.

## Discussion and conclusions

ENU mutagenesis applied to mice aims at generating mutant lines to obtain mouse models for interited human diseases. Subsequently, genomic variants are used in database searches to pinpoint candidate genes whose function may be related to the observed phenotype. After unraveling the genomic impact of mutagenesis on the murine phenotype, these mice may serve as model system for comparable human phenotypes.

Analysis of these genomic variants starts with the comparison of the sequence of the mutated mouse with a murine reference sequence. This reference sequence, in the case of mouse the assembly by NCBI and the Mouse Genome Sequencing Consortium, mm9 [10], represents a theoretical consensus sequence of an inbred wild type mouse, integrating sequence information from individuals and different strains. As a consequence, the bulk of the genomic variants observed in the course of such a comparison originates from natural differences between individual mice and mouse strains and the reference rather than from the mutagenesis itself. These variants have to be excluded since they are most likely irrelevant for the phenotype caused by the mutagenesis. In the present study, we were able to successfully filter out most of the strain-specific variation by using a closely related reference genome sequence such as C3H/HeJ instead of the common mm9 one. As a consequence, we obtained more specific mappings and reduced the number of sequence variants due to the evolutionary distance between C3H/HeJ and mm9.

An important contribution towards the selection of mutated candidate genes was the prior information we had for each of the mutant lines, in particular the mode of inheritance.

Linkage information, where available, was an additional factor to restrict the results of the variant analysis to a certain genomic region in 3 lines.

Since multiple unrelated mutant lines of the same strain background were included in the analysis, the set of the remaining lines served as filter for private variants in each one of the lines. The present set of 8 mutant lines shows that increasing the number of individuals from a given line helps to reduce the number of candidate positions complying with the expected genotype.

Looking only at a single line, e.g. Line 1, and selecting only exonic variants with homozygous variant genotype, we would have found 6369 positions instead of 1 position when using at least one of the other lines with 4 individuals (Line 2, Line 3, Line 4) as filter.

Similarly, when using at least one of the other lines with 2 individuals (Line 5, Line 6, Line 7, Line 8) as filter, we reduce to a maximum of 2 positions for Line 1.

Only positions with the required genotype in all individuals of the line were kept; thus the number of individuals per line also has an impact on the efficiency of filtering: In lines where 4 individuals were available, only 1 to 3 exonic variants remained after filtering, whereas in lines with only 2 individuals, the numbers range from 10 to 23. Although it might be possible to further improve the filtering by using other sources of annotations, it might not always be practical or necessary. For example, mm9 dbSNP positions could be lifted over to C3H/HeJ by aligning the surrounding sequence, but our tests showed a lower sensitivity and precision than with the exon lift-over.

Since ENU mutagenesis is typically executed at large scale, yielding tens of phenotypically selected lines, variant analysis of many lines in one batch is expected to yield very selective results, combining the benefits of both setting apart the causative random mutation in a single line from a background population and of having a good number of individuals per line (4 or more) available for genotyping.

Concluding, we have demonstrated that to align the sequencing reads to the available genome reference that is closest to the mouse strain used, in combination with exon lift-over for exon and gene annotation is the most specific direct path to discovering causative variants and novel gene-phenotype associations. We believe the approach described in this paper could be applied by most researchers dealing with mutagenised mice or other organisms with very close non-annotated genome references.

## Methods

### Ethics statement

The use of animals was in accordance with the German Law of Animal Protection and the tenets of the Declaration of Helsinki; it was approved by the Government of Upper Bavaria under the registration number 55.2-1-54-2532-126-11.

### Mutagenesis and breeding of mouse strains

ENU mutagenesis was performed in male C3H mice by intraperitoneal injections of 90 mg/kg ENU in three weekly intervals. Injected males (G0) were mated with C3H wild type female mice. Litters born later than hundred days after injection (F1 generation) were phenotyped to guarantee that they were derived from mutagenised sperm. For the dominant trait, F1 mice with a phenotype were mated with wild mice and in case of a Mendelian deviation of the phenotypes in at least twenty mice a new line was confirmed and given internal lab codes. To isolate recessive mutations, a two-step breeding scheme was used. Male F1 mice without any obvious phenotype were mated with wild type mice, female mice derived from these matings (G2) were mated with the F1 father. Twenty mice per gender of the offspring (G3) were phenotyped and the Mendelian rate of inheritance tested. For maintenance breeding new mouse lines were bred for at least five generations and archived by frozen sperm.

### DNA isolation and sequencing

The sample preparation for selected mouse gDNA regions capturing was performed using baits developed in collaboration with Roche-Nimblegen (Nimblegen SeqCap Mouse - mm9 beta2 capture kit [7]) and according to Nimblegen protocol for Illumina Paired-end Sequencing and also for Illumina TruSeq paired-end sequencing.

In brief, 3.0 μg of genomic DNA from murine spleens was sheared on a Covaris™ E220 to fragment size of 150-500 bp and size selected. For the Illumina Paired-end Sequencing protocol the fragment size of 300-450 bp was reached by agarose gel size selection. For the Illumina TruSeq paired-end sequencing protocol the fragments were size selected by AMPure XP beads to reach the fragment sizes of 250-500 bp. Fragmented DNA was end-repaired, adenylated and ligated to Illumina specific paired-end adaptors (for TruSeq paired-end protocol to Illumina indexed adaptors). The DNA with adaptor-modified ends was pre-capture amplified (9 cycles, Phusion™ High Fidelity PCR Master Mix and appropriate PCR primers PE1 and PE2 or PCR Primer cocktail resp.). The DNA fragments enrichment product quality was controlled on the Agilent 2100 Bioanalyzer with the DNA 7500 assay and 1.0 μg was hybridized to mouse Exome Library for 72 hrs on 47°C (Applied Biosystems 2720 Thermal Cycler). The hybridization mix was washed with wash buffers of different stringency in the presence of magnetic beads (Streptavidin Dynabeads, Life Technologies) at 47°C and the eluate was post-capture PCR amplified (18 cycles) with Illumina PCR primers appropriate to the kit used. The final library size and concentration was determined on Agilent 2100 Bioanalyzer 7500 chip. Each library was sequenced on one lane of a Genome Analyzer IIx (Illumina, Inc.) following the manufacturer’s protocol, in paired end mode with a read length of 2 × 76bp.

### Alignment of exome sequences

76 base paired end reads were aligned to the mm9 standard mouse reference and to the genome sequence of mouse strain C3H/HeJ as provided by Sanger [9], using GEM [11] and BFAST [12], outputting bam files [13]. The actual fasta sequence of the version of the C3H/HeJ reference is available upon request.

### Variant calling

Variant calling was performed using samtools and bcftools (version 0.1.18) [13] with default parameters.

### Exon and gene lift-over

The DNA sequences, Ensembl Exon IDs, Emsembl Gene IDs, Associated Gene Names and Chromosome Names were downloaded via Ensembl Biomart Structures [14] for *Mus musculus* version mm9. Each of these exon sequences was submitted to MegaBLAST (BLAST+ version 2.2.25) [15] against the C3H/HeJ genome sequence, allowing for only one hit per sequence to be output. For each hit, the start and end coordinates on the C3H/HeJ sequence were extracted along with the exon annotations, considering the directionality of the gene on the genome, and saved in Browser Extendible Data (BED) format [16]. In detail, coordinates for 5 types of genome annotations were extracted: The actual exonic regions were annotated with Ensembl Exon ID, its Ensembl Gene ID and its Associated Gene Name. Additionally, coordinates for entire genes were obtained using the outermost exon start and end positions from the first and last exon of a gene, respectively; these coordinates, which span both exonic and intronic regions of the gene, were annotated with Ensembl Gene ID and its Associated Gene Name, as well. These annotations were then incorporated in the vcf files using vcftools [17].

### Variant filtration

Many variant filtration strategies start by excluding positions that have been described previously and listed in public databases, such as dbSNP [18]. Because we used an alternative reference, we were not able to filter out positions from dbSNP (which uses the coordinates of the standard reference). Instead, we used accessory information from the experimental setup for filtering:

Based on the inheritance model for each line, we selected for positions that have the expected genotype (homozygous alternative for recessive and heterozygous for dominant) in all individuals of a line while all other lines are homozygous reference. Subsequently, we additionally selected for positions inside genes or exons. As in [1], private variants inside a gene that holds more than one variant were excluded, since it is highly unlikely, that ENU mutagenesis hit the same gene twice. We also highlighted those variants which show the predominant ENU-induced base changes in phenotype-based screens, namely T > C, A > G, T > A or A > T [19], as well as variants in regions previously confined in SNP arrays for each strain.

### Experimental evaluation of candidate positions

For linkage analysis, the mutation was outcrossed on the C57BL/6J strain according to the dominant or recessive breeding strategy, respectively. DNA extraction of tail clips samples from hybrid mice was performed as already described [20]. We used a panel of 158 genomic markers evenly distributed over the genome for SNP analyses by MALDI-TOF technology supplied by Sequenom (San Diego, CA, USA). We developed the internal MyGenotype database for statistical SNP data analysis [21]. By SNP analysis we obtained a genomic 30 MB region for further analysis.

Automated DNA extraction from tail clips used for candidate gene analysis was performed with the DNeasy Tissue Kit (Qiagen, Hilden, Germany). Sanger type DNA sequencing was done using an ABI 310 capillary sequencer (Applied Biosystems, Darmstadt, Germany) as already described [22].

## Availability of supporting data

The raw sequencing read files (FASTQ) for the 8 mutant mouse lines on which the results of this article are based have been made available at the ENA, with study accession number PRJEB8962 at http://www.ebi.ac.uk/ena/data/view/PRJEB8962.

## Abbreviations

ENU: N-Ethyl-N-Nitrosourea
MB: Megabase of the genome
SNP: Single nucleotide polymorphism
MALDI-TOF: Matrix-assisted laser desorption/ionization time-of-flight
